# A new species of *Hornylia* Wygodzinsky (Hemiptera, Heteroptera, Reduviidae, Emesinae) from Thailand

**DOI:** 10.3897/zookeys.917.46887

**Published:** 2020-03-09

**Authors:** Zhuo Chen, Hu Li, Wanzhi Cai

**Affiliations:** 1 Department of Entomology China Agricultural University Beijing China; 2 and MOA Key Lab of Pest Monitoring and Green Management, College of Plant Protection, China Agricultural University, Beijing 100193, China China Agricultural University Beijing China

**Keywords:** Emesinae, *
Hornylia
*, Metapterini, new species, Oriental Region, taxonomy

## Abstract

*Hornylia obtusipetala***sp. nov.** from eastern Thailand is described and illustrated. This new species is the second representative of the genus *Hornylia* Wygodzinsky, 1966. A key to species of *Hornylia* is presented. The relationship with allied genera and distribution of *Hornylia* is briefly discussed. *Hornylia* is recorded from Thailand for the first time.

## Introduction

Emesinae, or thread-legged assassin bugs, have long intrigued scientists not only because of their bizarre looking (Dohrn 1860, 1863; Wygodzinsky 1966), but also for their interesting web-dwelling (Distant 1915; Wignall and Taylor 2010; Soley et al. 2011) and cave-living (Kemp and China 1924; Gagné and Howarth 1975; Ribes et al. 1998; Pape 2013; Chłond et al. 2018) habits. The tribe Metapterini Stål, 1874 is the most speciose group within Emesinae, with 29 genera and approximately 280 species are described (Maldonado-Capriles 1990; Popov 1991). Metapterini are characterized by the conspicuous basal process of posteroventral series of fore femur, and high proportion of genera with wing polymorphism (Wygodzinsky 1966; Castro-Huertas et al. 2019). However, recent phylogenetic analyses based on morphology of genitalia (Castro-Huertas et al. 2018) and fore leg (Castro-Huertas et al. 2019) consistently indicate that Metapterini are paraphyletic with respect to Deliastini Villiers, 1949, showing that our knowledge on the evolution of Metapterini remains far from complete.

The genus *Hornylia* Wygodzinsky, 1966 is one of ten monotypic genera among Metapterini. It was established by Wygodzinsky (1966) in his epic monograph on Emesinae. The type species, *Hornylia nalanda* Wygodzinsky, 1966, was described based on a single male specimen from Nalanda, Ceylon (now Sri Lanka) (Wygodzinsky 1966). No more information has been published since then except for being documented in the worldwide catalogue (Maldonado-Capriles 1990), and the female of *Hornylia* is still unknown (Castro-Huertas et al. 2018), making this genus somewhat enigmatic.

During our recent examination of emesine specimens deposited in the Entomological Museum of China Agricultural University, Beijing, China (CAU), a male specimen of *Hornylia* from eastern Thailand was discovered. It differs from *H. nalanda* in several characters and warrants description as a new species.

## Materials and methods

Type material is preserved in the Entomological Museum of China Agricultural University, Beijing, China (CAU). Male genitalia were soaked in hot 10% KOH solution for approximately five minutes to remove soft tissue, rinsed in distilled water, and dissected under a Motic binocular dissecting microscope. Dissected genitalia were placed in a vial with glycerin and pinned under the corresponding specimen after examination. Photographs were all taken by Canon 7D Mark II digital camera with Canon micro lens EF 100 mm and MP-E 65 mm for habitus and an Olympus BX51 microscope for dissected body parts. Helicon Focus version 5.3 was used for image stacking. Measurements were obtained using a calibrated micrometer. Morphological terminology mainly follows Wygodzinsky (1966), but the term “rostrum” is replaced with “labium”. The visible labial segments are numbered as II to IV, given that the first segment is lost or fused into the head capsule in most Reduviidae (Weirauch 2008; Schuh et al. 2009).

## Taxonomy

### Genus Hornylia

Taxon classificationAnimaliaHemipteraReduviidae

Wygodzinsky, 1966

745A49F1-91D1-5B76-972A-CCC77DF71FAC

Hornylia Wygodzinsky, 1966: 494. Type species by original designation: Hornylia nalanda Wygodzinsky, 1966.

#### References.

Maldonado-Capriles, 1990: 131.

#### Diagnosis.

Apterous; body surface dull, granulated; anteocular part longer than postocular part; interocular furrow strongly curved backwards at its midpoint, reaching far behind level of posterior margin of eyes; fore femur with three series of spiniferous processes, and each process bearing a short spine apically; posteroventral series beginning very close to base of femur, composed of several large and numerous small processes; anteroventral series widely interrupted at base; fore tarsus not segmented, with a single claw on its apex.

#### Diversity and distribution.

Two species, occurring in the Oriental Region.

### Hornylia obtusipetala sp. nov.

Taxon classificationAnimaliaHemipteraReduviidae

7F933C29-A767-58B1-820D-5BEADACF5EDF

http://zoobank.org/996100A3-B8D6-43F3-9F8F-A80B0131A035

Figs 1–3 , 4–11 , 12–22

#### Diagnosis.

Body length 10.98 mm; apex of labial segment II not reaching level of anterior margin of eyes (Fig. 5); anteroventral series of fore femur consisting of about seven medium-sized processes, posteroventral series consisting of ca. four large- and two medium-sized processes (Figs 6, 7); mid and hind femora each with one indistinct, brown medial annulus and two distinct, blackish brown annuli, one beyond middle and another subapically (Fig. 8); ventral surface of abdomen brown, mottled with blackish brown (Figs 3, 8, 10); parameres expanded and blunted apically, with a sharp subapical process (Figs 10–12, 18–20).

#### Description.

**Figures 1–3.**
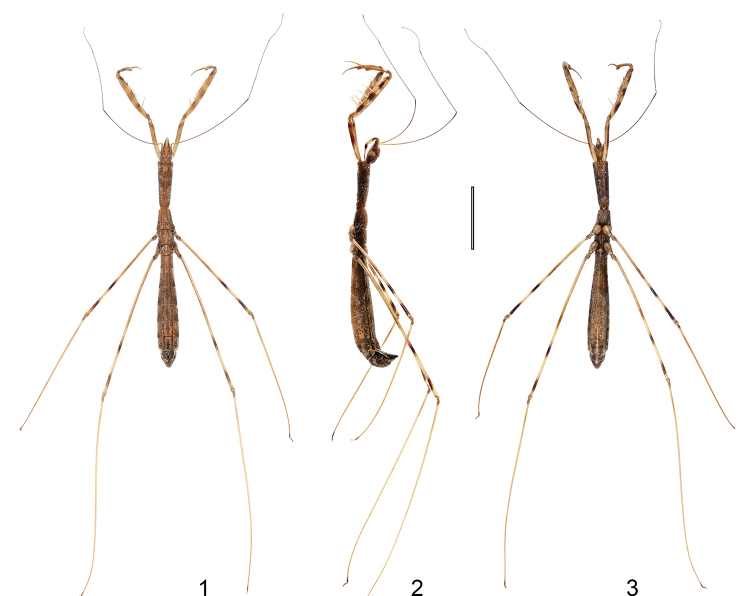
*Hornylia obtusipetala* sp. nov., male, holotype, habitus **1** dorsal view **2** lateral view **3** ventral view. Scale bar: 3.00 mm.

**Figures 4–11.**
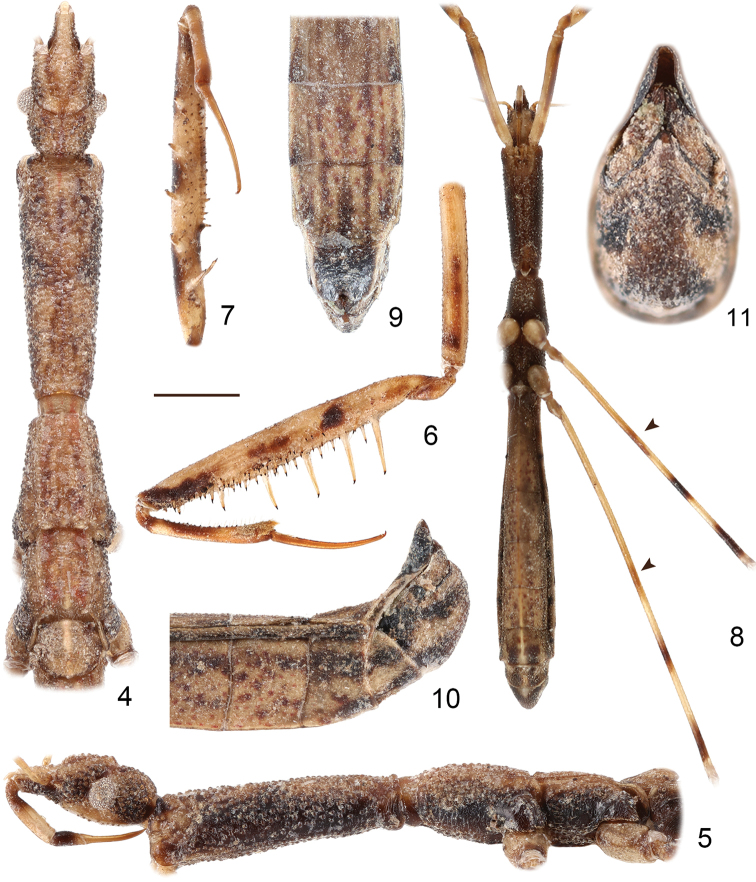
*Hornylia obtusipetala* sp. nov., male **4, 5** head, thorax and base of abdomen, antennae and legs removed **6** left fore leg **7** right fore femur, tibia and tarsus **8** body with fore coxae, femora of left mid and hind legs, arrows indicating the indistinct annuli on ventral surface of mid and hind femora **9–11** apex of abdomen **4, 9** dorsal view **5, 6, 10** lateral view **7, 8** ventral view **11** caudal view. Scale bars: 0.75 mm (**4–7, 9, 10**); 1.50 mm (**8**); 0.375 mm (**11**).

**Figures 12–22.**
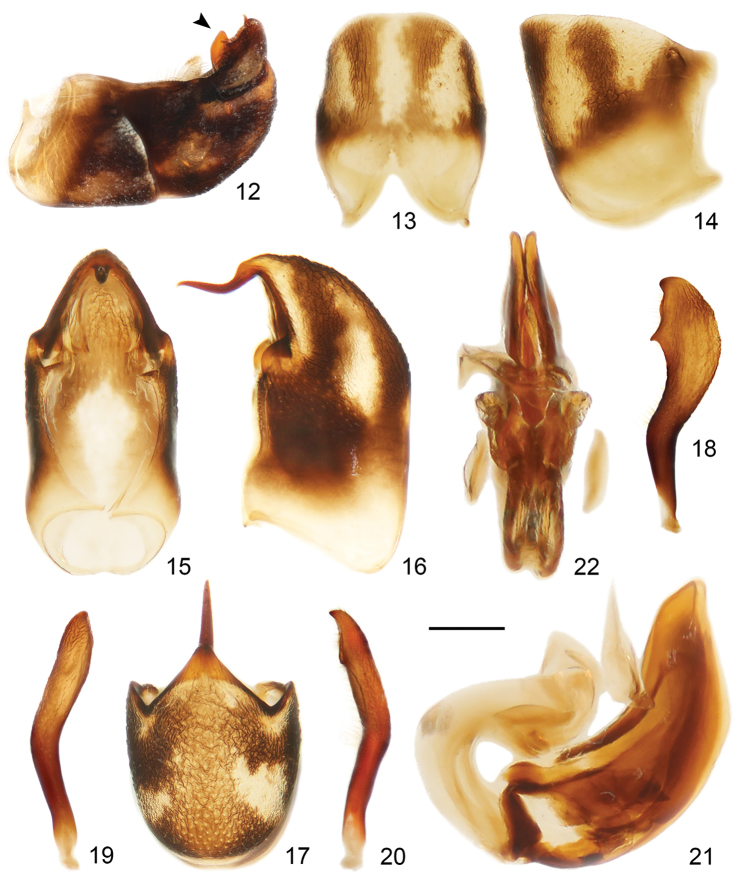
*Hornylia obtusipetala* sp. nov., male **12** abdominal segment VIII and genitalia, the arrow points to the apex of phallus **13, 14** abdominal segment VIII **15–17** pygophore **18–20** paramere **21, 22** phallus **12, 14, 16, 18, 21** lateral view **13, 15, 20, 22** dorsal view **17** caudal view **19** ventral view. Scale bars: 0.25 mm (**12**); 0.375 mm (**13–19**); 0.50 mm (**20–21**).

Apterous male. ***Coloration***: Body generally yellowish brown (Figs 1–3). Head (Figs 4, 5): lateral surface as well as gena blackish brown; postocular part slightly mottled with black; eyes silvery; antennae reddish brown, base of first segment pale brown, darkening toward its apex gradually; clypeus and labrum light brown; labium somewhat shiny, labial segment II light brown on apical 2/3, segment III (except apex) dark brown. Prothorax (Figs 4, 5): lateral surface and tubercle of each anterolateral angle blackish brown, ventral surface reddish brown. Meso- and metathorax (Figs 4, 5) with blackish brown lateral surface and reddish brown ventral surface. Fore coxa light brown on basal half and brown on apical half, with a dark brown subapical patch on inner and outer surfaces; fore trochanter brown as apical half of coxa; fore femur light brown, with subbasal, medial, and apical patches dark brown, spiniferous processes light yellowish brown, with their apical spines black; fore tibia light brown as general color of femur, with base, medial patch and apex brown, denticles on ventral surface black; fore tarsus brown, shiny (Figs 6, 7). Mid and hind femora (Figs 1–3, 8) light yellowish brown, with a brown, indistinct medial annulus and two blackish brown, very distinct annuli, one situated beyond middle, and another subapically; mid and hind tibiae (Figs 1–3) light yellowish brown with their bases and apexes brown; mid and hind tarsi uniformly brown. Abdomen (Figs 1–3, 9, 10): tergites with an obscure, nearly disrupted medial stripe and two pairs of lateral brownish stripes, apical half of tergite VII blackish brown (Fig. 9); dorsal laterotergites (Fig. 9) yellowish brown as general body color, their posterior halves reddish brown to dark brown, posterolateral angles blackish brown; ventral laterotergites (Fig. 10) blackish brown, with outer margin yellowish brown; sternites with dark brown suffusion and a pair of lateral brownish stripes ; sternite VII (Fig. 10) and segment VIII (Figs 10, 12–14) each with two pairs of blackish brown bands; pygophore (Figs 10–12, 15–17) blackish brown and suffused with yellowish brown.

***Structure***: Body elongate. Surfaces of head, thorax and abdomen conspicuously granulated (Figs 1–5). Body sparsely clothed with very short, decumbent setosity, difficult to observe; first and second (except apical portion) antennal segments sparsely clothed with short setae; apical portion of second antennal segment as well as third and fourth antennal segments densely clothed with decumbent, short pubescence; dorsum of fore trochanter with a pair of erect, long setae; fore femur and fore tibia with numerous erect, long setae (Fig. 6); apex of fore tibia and base of fore tarsus with dense, decumbent, long golden setae (Fig. 7); femora and tibiae of mid and hind legs densely clothed with short, decumbent setae; mid and hind tarsi densely clothed with short pubescence.

Head (Figs 4, 5) porrect forwardly, 1.76 times as long as wide across eyes; anteocular part 2.13 times as long as postocular part; postocular part strongly granulated on dorsum; interocular space 3.50 times as wide as a single eye in dorsal view; eyes rather small, protruding laterally in dorsal view, far remote from dorsal and ventral outlines of head in lateral view; antennae 0.59 times as long as body length, with first segment longest and third segment shortest; labrum smooth; labium as shown in Fig. 5, labial segment II longest, strongly curved at base, segment III shortest, slightly swollen, reaching anterior margin of eyes, segment IV gradually tapering. Prothorax (Figs 4, 5) subcylindrical, 1.54 times as long as head, and 4.17 times as wide as its greatest width; pronotum divided vaguely into anterior and posterior lobe, with anterior and posterior margins concave, posterior lobe extremely short and indistinct; posterior margin of prosternum largely concave, emarginated. Meso- and metanota (Figs 4, 5) carinated longitudinally along midportion, mesonotum 0.36 times as long as pronotum, metanotum 0.34 times as long as pronotum. Fore legs (Figs 6, 7) stout; fore coxa cylindrical, 0.68 times as long as fore femur; fore trochanter simple, unarmed in venter; anteroventral series of fore femur composed of about seven medium-sized and 20 small-sized processes; posteroventral series composed of about four large-sized, two medium-sized, and eleven small-sized processes, basal most process longest, distinctly longer than distance between basal most process and base of fore femur; accessory series composed of ca. 13–15 small-sized processes arranged irregularly; fore tibia short, 0.45 times as long as fore femur, ventrally with 10–12 strongly sclerotized denticles; fore tarsus 0.80 times as long as fore tibia, slightly curved, ventrally with a row of decumbent, knifelike setae. Mid and hind legs (Figs 1–3, 8) slender; mid and hind tibiae 1.25 and 1.64 times as long as respective femora, hind tibia slightly shorter than body length; mid and hind tarsi minute, apically with a pair of sickle-like claws. Abdomen (Figs 1–3, 8) elongate, 6.14 times as long as its greatest width, with a medial longitudinal ridge on ventral surface; abdominal tergite VII (Figs 9–11) projected posteriorly, apically rounded, warping upwardly, covering most part of pygophore; segment VIII (Figs 10, 12–14) distinctly exposed in lateral view, anteromedial margin strongly concave, posteromedial margin nearly straight.

***Male genitalia***: At rest as shown in Fig. 12. Pygophore (Figs 12, 15–17) elongate oval, anterior dorsal sclerotization narrow, insertion of paramere slightly produced; posterosuperior process (Figs 16, 17) elongate spine-like, bent near base, apex sharp, slightly curved. Parameres (Figs 18–20) broad, covered with simple setae, apical half expanded, apex blunted; subapical projection (Fig. 18) acute; margin between apex and subapical projection emarginated. Phallus as in Figs 21 and 22: articulatory apparatus thickened, strongly curved; basal plates separate; pedicel very short; phallosoma divided into two lobes, strongly sclerotized, apex blunt.

***Measurements*** [in mm, male (*N* = 1)]. Length of body 10.98; length of head 1.30; length of anteocular part 0.49; length of postocular part 0.23; width across eyes 0.74; interocular space 0.47; length of antennal segments I–IV = 3.20, 2.25, 0.37, 0.71; length of labial segments II–IV = 0.38, 0.15, 0.32; length of anterior pronotal lobe 1.91; length of posterior pronotal lobe 0.09; width of anterior pronotal lobe 0.48; width of posterior pronotal lobe 0.32; length of mesonotum 0.72; length of metanotum 0.68; length of fore coxa, trochanter, femur, tibia, tarsus (without claw) = 1.29, 0.29, 1.91, 0.87, 0.70; length of mid femur, tibia, tarsus = 5.41, 6.76, 0.24; length of hind femur, tibia, tarsus = 6.20, 10.15, 0.27; length of abdomen 3.81; maximum width of abdomen 0.62.

#### Type material.

***Holotype*** (male): Thailand, Chanthaburi, Khao Soi Dao, 25.xii.2007, leg. W. Sakchoowng (CAU).

#### Etymology.

The specific epithet is derived from Latin *obtus*- (meaning obtuse or blunt) and -*petala* (meaning petal), referring to the apically expanded and blunted parameres of the new species.

#### Distribution.

Thailand (Chanthaburi).

## Discussion

### Comparative notes

*Hornylia obtusipetala* sp. nov. can be distinguished from *H. nalanda* by its considerably larger size and several different morphological characters which are discussed. The apex of labial segment II of the new species is far from the level of anterior margin of eyes (Fig. 5), while in *H. nalanda* it reaches the level of anterior border of eyes. The anteroventral series of the fore femur of *H. obtusipetala* sp. nov. consists of ca. seven medium-sized processes (Figs 6, 7), while in *H. nalanda* the processes number is five; the posteroventral series consists of four large-sized and two medium-sized (one situated between basal most and second basal large-sized processes, another subapically) processes in *H. obtusipetala* sp. nov. (Figs 6, 7), but have six large-sized processes in *H. nalanda*. Third, mid and hind femora of *H. obtusipetala* sp. nov. have three dark brown annuli (medial one brown and indistinct, the other two blackish brown and distinct, one situated beyond middle, one subapically) (Figs 1–3, 8), while in *H. nalanda* there are two annuli (one situated beyond middle, indistinct, and another subapically, very distinct). Finally, the ventral surface of the abdomen is yellowish brown and suffused with dark brown in the new species (Figs 3, 8), while in *H. nalanda* it is piceous on ventral surface.

The shapes of the parameres greatly differ between *H. obtusipetala* sp. nov. and *H. nalanda*: in the former, the parameres are broad, apically expanded and blunted, with a conspicuous subapical process (Figs 18–20); while in the latter, the parameres are slender, apically sickle-shaped, and without any subapical process. Wygodzinsky (1966) mentioned that phallosoma of *H. nalanda* is sclerotized dorsally, whereas the phallosoma of *H. obtusipetala* sp. nov. is entirely strongly sclerotized (Figs 21, 22). Moreover, the shape of abdominal segment VIII, pygophore, and capitate processes of the phallus of *H. obtusipetala* sp. nov. are quite different from *H. nalanda*.

Species of the genus *Hornylia* can be distinguished with the key below.

### Key to species of *Hornylia* Wygodzinsky, 1966

**Table:** 

1	Body length ca. 8 mm; labial segment II reaching level of anterior margin of eyes; anteroventral series consisting of five medium-sized processes; posteroventral series consisting of six large-sized processes; mid and hind femora with one distinct annulus; parameres slender, apically sickle-shaped, without subapical process	***Hornylia nalanda* Wygodzinsky**
–	Body length ca. 11 mm; labial segment II not reaching level of anterior margin of eyes; anteroventral series consisting of seven medium-sized processes; posteroventral series consisting of four large-sized and two medium-sized processes; mid and hind femora with two distinct annuli; parameres broad, apically expanded and blunted, with a sharp subapical process	***Hornylia obtusipetala* sp. nov.**

### Genera related to *Hornylia*

The Oriental genus *Hornylia* seems to be related to *Bobba* Bergroth, 1914 (Afrotropical, 5 spp.), *Bargylia* Stål, 1866 (Australasian, 6 spp.), and *Leaylia* Wygodzinsky, 1966 (Australasian, 1 sp.) due to their similar appearance and several shared morphological characters: the anteocular part distinctly longer than postocular part; antennal insertion situated before the middle of the anteocular part but relatively far from apex of head; labial segment II at least twice as long as segment III; ventral spiny region of fore femur occupying at least half of the length of fore femur; fore tarsus with one segment, with decumbent, strongly sclerotized setae on ventral surface; mid and hind claws generally medially incised, without other projections; parameres without sensory spines (Wygodzinsky 1966; Rédei 2007, 2013; Tatarnic and Cassis 2011). However, no comprehensive phylogenetic hypothesis is available for Emesinae, and only one species (*Bargylia longinota* Wygodzinsky, 1956) is included in recent morphology-based phylogenetic analyses of Metapterini (Castro-Huertas 2018, 2019). Therefore, a strict cladistic analysis is needed to clarify the generic relationships within this group.

### Distribution of *Hornylia*

Prior to the discovery of *H. obtusipetala* sp. nov. from Thailand, the single representative of the genus, *Hornylia nalanda*, was only known from its type locality in Ceylon (now Sri Lanka). The discovery of the new species described here extends the distribution range of *Hornylia* to the mainland Southeast Asia. It is possible that more *Hornylia* species will be discovered on mainland Asia and adjacent islands in the future.

Emesinae are widely distributed around the world, but exhibit high diversity on isolated islands, with numerous endemic island taxa (Wygodzinsky 1966; Villiers 1979; Rédei and Tsai 2010; Tatarnic and Cassis 2011; Ishikawa and Miyamoto 2012). This distribution pattern frequently presents at the species level within Emesinae, but also occurs at some generic level taxa, e.g., *Atisne* Wygodzinsky, 1966 with two species endemics to Lord Howe Island (Tatarnic and Cassis 2011), and *Saicella* Usinger, 1958 and *Nesidiolestes* Kirkaldy, 1902 restricted to the Hawaiian Islands (Polhemus 2000). However, the majority of island-endemic genera are monotypic, with a single or few specimens collected only on a single occasion (Wygodzinsky 1966; Wall and Cassis 2003). Recent studies on the genus *Stenorhamphus* Elkins, 1962 revealed that the distribution range of this group is much wider than originally known (Smith et al. 2019). Similarly, the “Borneo endemic” genus *Chinemesa* Wygodzinsky, 1966 (5 spp.) was recently discovered on mainland Asia (Chen et al. 2020). It is obvious that our knowledge of the distribution of these island-endemic taxa is very rudimentary, and the discovery of more species, as well as robust phylogenetic analyses in the future will help to better understand the biogeography of Emesinae.

## Supplementary Material

XML Treatment for Hornylia

XML Treatment for Hornylia obtusipetala
